# Acute and Sublethal Effects of Deltamethrin Discharges from the Aquaculture Industry on Northern Shrimp (*Pandalus borealis* Krøyer, 1838): Dispersal Modeling and Field Investigations

**DOI:** 10.1021/acs.est.2c07459

**Published:** 2023-02-24

**Authors:** Maj Arnberg, Gro Harlaug Refseth, Ian John Allan, Maura Benedetti, Francesco Regoli, Luca Tassara, Kjetil Sagerup, Magnus Drivdal, Ole Anders Nøst, Anita Evenset, Pernilla Carlsson

**Affiliations:** † Akvaplan-niva, Pirsenteret, Havnegata 9, 7010 Trondheim, Norway; ‡ Norwegian Institute for Water Research (NIVA), Økernveien 94, 0579 Oslo, Norway; § Department of Life and Environmental Sciences, Polytechnic University of Marche, 60 131 Ancona, Italy; ∥ Akvaplan-niva, Fram Centre, Hjalmar Johansens Gate 14, 9007 Tromsø, Norway; ⊥ Norwegian Institute for Water Research (NIVA), Fram Centre, Hjalmar Johansens Gate 14, 9007 Tromsø, Norway; # National Future Biodiversity Center (NFBC), Palermo, Italy

**Keywords:** delousing agents, pesticides, passive sampling, silicon rubber, lethal dose, Alpha Max, salmon fish farming

## Abstract

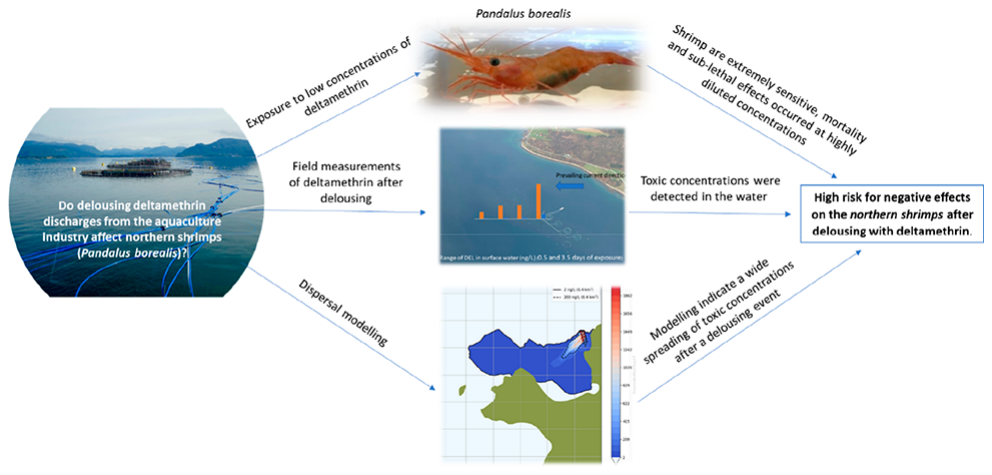

Pharmaceutical deltamethrin (Alpha Max), used as delousing treatments in aquaculture, has raised concerns due to possible negative impacts on the marine environment. A novel approach combining different scientific disciplines has addressed this topic. Acute (mortality) and sublethal effects (i.e., fitness, neurological, immunological, and oxidative responses) of exposure of northern shrimp (*Pandalus borealis*) were studied in laboratory experiments. Passive water sampling combined with sediment analyses revealed environmental concentrations. Finally, dispersal modeling was performed to predict environmental concentrations. Ecotoxicological analyses showed mortality in shrimp after 1 h of exposure to 2 ng L^–1^ (1000-fold dilution of treatment dose), revealing a high sensitivity to deltamethrin. Sublethal effects included induction of acetylcholinesterase and acyl CoA oxidase activities and oxidative impairment, which may be linked to neurotoxic responses. Field concentrations of 10–200 ng L^–1^ in water (100 m from the pens) and <LOD-0.19 ng g^–1^ dw in sediment (0–400 m from pens) were measured. Ecotoxicological values were compared with measured and modeled concentrations. They showed that concentrations higher than those causing mortality could be expected up to 4–5 km from point of release, in an area of 6.4 km^2^, with lethal concentrations remaining up to 35 h in some areas. Hence, the study demonstrates that there is a considerable risk for negative effects on the ecologically and commercially important shrimp.

## Abstract 2

Environmental effects of chemicals in the aquaculture industry can be documented by multidisciplinary approaches to inform policy and stakeholders.

## 1 Introduction

Aquaculture is a worldwide and exponentially growing industry. Norway alone produced salmon (*Salmo salar* (Linnaeus, 1758)) worth €6.6 billion in 2019 and is one of the world’s largest fish farming countries.^1^ A major challenge in aquaculture is the infestation of salmon by the salmon louse (*Lepeophtheirus salmonis* (Krøyer 1837)) that causes economical loss, fish welfare impairment, and impacts on wild fish stocks. Several techniques are used to mitigate this problem, including mechanical, biological, or pharmaceutical methods.

The current study focuses on a delousing pharmaceutical agent (deltamethrin) that is applied as a bath treatment to control sea lice within farm cages.^2^ Bath treatment pharmaceuticals are added directly to the pens that are covered with a tarpaulin or to treatment water in well boats. After the treatment of salmon, the water is released directly to the surrounding marine environment,^3−5^ and large volumes containing the delousing agents are discharged to the marine environment in Norway each year.

The release of bathing chemicals (hydrogen peroxide, azametiphos, delta- and cypermethrin) has only been regulated in Norway since 2019, when releases within a 500 m distance of known shrimp areas were prohibited.^6^ However, oceanographic modeling results have predicted that these chemicals spread in various directions and at harmful concentrations, several kilometers away from the discharge point depending on season, ocean currents, and local conditions.^7−12^ Few measurements of delousing agents have been performed in the field, but modeling results clearly indicate that today’s regulation is not sufficient.

Nontarget marine organisms living in areas with salmon aquaculture activity can be exposed repeatedly to a combination of chemicals. One of the delousing bath chemicals that has recently caused concern for its high toxicity is the pyrethroid (a synthetic insecticide) deltamethrin (trade name Alpha Max hereafter only referred to as deltamethrin). 10–158 kg year^–1^ of the active substance has been used in salmon aquaculture in Norway since the approval in 2006.^13,14^

Several studies have demonstrated the high toxicity of deltamethrin to different marine species.^9,15−17^ In general, crustaceans are most sensitive, therefore concerns have been raised, especially for ecologically and commercially important crustacean species, such as the northern shrimp (*Pandalus borealis*). In a recent study, high mortality was observed in egg-bearing northern shrimp exposed to low concentrations of pharmaceuticals used as bath treatments, calling for future studies on potential sublethal effects at even lower exposure levels.^15^ Bamber et al.^18^ demonstrated that low concentrations of deltamethrin exposure triggered behavior alterations in northern shrimp with an immediate increase in swimming activity and then reduced intensity, leaving all shrimp either moribund or dead after 22 h. The mode of action of deltamethrin is through interference of neuron signal transmission by disruption of the sodium and potassium channels leading to paralysis and death in organisms (e.g., refs (9, 15, 19−22)). Other bath treatments are shown to be acetylcholinesterase inhibitors that cause paralysis in the sea lice, following accumulation of the neurotransmitter acetylcholine^15^. Therefore, behavioral activity together with more sublethal biomarker responses (e.g., acetylcholinesterase activity) may serve as ideal end points to measure the effects of exposure to diluted solutions of delousing agents.

To detect possible impact zones of deltamethrin to nontarget organisms (e.g., northern shrimp), there is a need to investigate how delousing chemicals spread in the environment and to document field concentrations close to farms. Passive sampling can be a useful sampling method when contaminant concentrations in water are variable, e.g., in the case of a plume of contaminants. Passive sampling is based on the deployment *in situ* of devices capable of accumulating contaminants of interest over time.^23−25^ This allows the determination of time-integrated contaminant concentrations for the period of exposure. However, it is challenging to accurately sample and measure chemical spreading. Traditional water sampling measures a single point in time and space, and if samples are collected outside the dispersal plume, the measured concentrations will be quite different from those inside the plume. The direction of the plume will vary with wind and tidal current, and a method that measures concentrations for a longer period and thus from a larger water volume will be more appropriate to “detect” the actual plume. Sampling at an increasing distance from the pens will also make it gradually more difficult to “detect” the actual plume. Thus, oceanographic dispersal modeling is a good solution to provide a more detailed picture of the spreading of the chemicals further away from the fish farms.

The aim of the present study is to provide critical new knowledge through a multidisciplinary approach that can be used to assess the impact of deltamethrin in the marine environment. First, lab experiments on the northern shrimp were performed to document concentrations of deltamethrin causing the onset of acute and sublethal effects. Second, the proof of concept of passive sampling and analytical techniques allowed field measurements of deltamethrin in the vicinity of a fish farm. Third, dispersion modeling was used to predict how far potential harmful concentrations to shrimp can spread. Finally, all results were used to assess the risk for potential negative effects on northern shrimp after delousing with deltamethrin.

## 2 Material and Methods

### 2.1 Lab Experiments with Northern Shrimp

#### 2.1.1 Collection

Northern shrimp were collected with shrimp pots in fjords in northern Norway in October 2019 and acclimatized in the research laboratory until the start of the experiment (details can be found in the Supporting Information (SI)).

#### 2.1.2 Exposure Scenarios and Experimental Setup

Selected exposure concentrations were based on the sensitivity of adult shrimp to deltamethrin determined in recent experiments^15,16,18^ in addition to toxicity data reported for other crustacean species.^17,26^ The number and length of exposure pulses were based on environmentally relevant scenarios (Table S1) obtained in previous dispersal and toxicological studies.^15,16,26−28^ These studies indicated that pesticides diluted to 0.1% of the original treatment concentration (2 000 ng L^–1^) could persist for some hours at distances up to 2 km from the original point of discharge.^20^

The experiments were carried out in November 2019, and lethal and sublethal effects of deltamethrin were investigated. Shrimp were exposed to three short pulses (each lasting 1 h/day repeated for three consecutive days), followed by a post exposure period in clean seawater of 14 days. Four treatments were used in the experiment, Control (pulses of clean seawater), low (pulses of 0.0008 ng L^–1^ deltamethrin), middle (pulses of 0.04 ng L^–1^ deltamethrin), and high (pulses of 2 ng L^–1^ deltamethrin; Table S1).

All exposure experiments were conducted in 60 L flow-through tanks (SI, Figure S1). Shrimp were placed into the exposure tanks 48 h prior to exposure start for acclimation. Five replicate tanks with eight shrimp in each for each treatment, including control, were used (Figure S1). All experimental procedures used were approved by the Norwegian Animal Research Authority (FOTS), FOTS ID 20997.

#### 2.1.3 Effect Parameters

##### Mortality and Behavior

Shrimp were visually observed pre- and postexposure with regard to behavior, and the following classification was applied: shrimp standing (normal behavior), swimming activity, and lying on the side/loss of equilibrium. Animals were considered dead (and then decapitated) when there was a lack of reaction and/or when lying on their side. During the 14-day recovery period, shrimp behavior was observed once a day. After the final exposure, a subsample of five shrimp per replicate tank were killed by decapitation. Length (±1.0 mm) and total weight (±0.001 g) were recorded, and samples of gills, muscle, and hepatopancreas were frozen at −80 °C until further analyses. At the end of the recovery period, the remaining shrimp were sampled following the same protocol. Samples were shipped in a liquid-nitrogen dry shipper container to Polytechnic University of Marche (Italy) for biomarker analyses.

##### Sublethal Effects/Biomarker Analysis

A selection of sublethal end points related to, e.g., neurological impacts, lipid metabolism, and oxidative responses of shrimp were addressed in the study. Validated protocols were used to analyze the following parameters: acetylcholinesterase activity (AChE) in gills and muscle tissues to assess neurotoxicity; AcylCoA (acyl coenzyme A) oxidase activity (ACOX), involved in different aspects of lipid homeostasis in the digestive gland; antioxidant response and oxidative damage in digestive gland by total oxyradical scavenging capacity (TOSC assay toward peroxyl and hydroxyl radicals); and lipid peroxidation (malondialdehyde levels). The parameters described above were analyzed in tissues at the end of exposure (day 4) and at the end of the recovery period (day 14). Analytical methods are described in the SI.

### 2.2 Field Sampling of Sediment and Water

Two different aquaculture sites in northern Norway were chosen for case studies. At site 1, passive water sampling was carried out during delousing and sediment sampling 5–6 weeks later, while at site 2 only sediments were sampled one month after delousing. Both sites have a soft bottom and are of 50–150 m depth. Sampling stations were placed downstream from the prevailing current direction^29^ to ensure exposure to released treatment water after delousing.

#### 2.2.1 Evaluation of Passive Sampling Technique

Silicone rubber passive samplers (PAS) were selected for water sampling of delousing chemicals. PAS have previously been used for a wide range of compounds.^30−32^ For an accurate estimation of freely dissolved concentrations, polymer–water partition coefficients (*K*_pw_) are needed. These are generally measured in laboratory experiments.^33^ Uptake experiments with PAS were therefore conducted to establish the polymer–water partition coefficient (*K*_pw_) before deployment in the field. *K*_pw_ was measured for deltamethrin using the cosolvent method for two types of silicone rubber.^32−34^ Simultaneously, *K*_pw_ for the bath treatment chemical cypermethrin and the in-feed chemicals diflubenzuron and teflubenzuron were established (detailed methods in the SI).

#### 2.2.2 Deployment of Passive Samplers and Sediment Collection

##### Site 1

The delousing at site 1 took place in the pens during 3 days in early spring in 2020, and a total of 27 L of Alpha Max was used, which corresponds to 270 g of deltamethrin for the full treatment. The site had not been treated with deltamethrin since 2017.^35^

PAS made of AlteSil silicone rubber sheets (thickness: 0.5 mm, purchased from Altec, UK) were spiked with performance reference compounds (PRCs; details in the SI) prior to deployment. These non-naturally occurring compounds are used to estimate *in situ* contaminant exchange kinetics between water and silicone rubber. The samplers (A–E) were deployed 3 days before delousing, 15–120 m from the deloused pens at 3–5 m and 10–14 m depth at five sampling sites (*n* = 10). PAS were collected 3 days after the delousing ended (Table S2). Sampler A, situated inside a deloused pen, was lost, but samplers B–E were recovered and stored frozen in tin containers until analyses. Surface sediment samples (*n* = 10) were collected 5 weeks after the last delousing event at distances 0–500 m from the pens at 70–130 m depth with a Van Veen grab, and the 0–1 cm top layer was transferred to preburned (450 °C) glasses and kept frozen until analyses (Table S2). Only grabs with an undisturbed surface were approved for sampling.

##### Site 2

Delousing took place in the pens during winter 2019, and a total of 17.5 L of Alpha Max was used, which corresponds to 175 g of deltamethrin for the full treatment. The dose used was 1.5× the recommended dose, i.e., 3000 ng L^–1^ (personal communication, Aquaculture Company). There were two other delousing events with deltamethrin on this site in 2019, 12 months before our sampling campaign (whole area) and 6 months before (partial delousing). Surface sediment samples (*n* = 12, 0–1 cm) were collected 6 weeks after the last delousing at distances 0–300 m from the pens at 20–100 m depth.

#### 2.2.3 Chemical Analyses, Quality Control, and Calculations of Deltamethrin Concentration

Sample preparation, cleanup, and analyses of sediment and water samples are described in the SI. Control samples spiked with a known amount of deltamethrin and blanks were analyzed parallel with the field samples (details in SI). Calculations of the uptake of deltamethrin in PAS are based on the work of Rusina et al.^36^ and are described in the SI.

The limit of detection (LOD) was set to 3× the signal-to-noise ratio (S/N). Due to interference from the sediment and varying characters of sediment samples, LOD varied between 0.02 and 0.1 ng g^–1^ dw for site 1 samples and 0.1–0.5 ng g^–1^ dw for site 2 samples. LODs for PAS were 0.04 ng g^–1^ PAS, which equals 4 ng L^–1^ for 0.5 days of exposure and 0.6 ng L^–1^ for 3.5 days of exposure.

### 2.3 Oceanographic Modeling

#### 2.3.1 Hydrodynamic and Dispersion Model

For the modeling in this study, a random aquaculture location in northern Norway was chosen as a case study (Figure S4). To simulate the dispersion of deltamethrin after delousing in fish cages, the hydrodynamic model FVCOM (Finite Volume Community Ocean Model^37^) was coupled with a tracer model through FABM (Framework for Aquatic Biogeochemical Models^38^). Due to its unstructured grid, FVCOM is well suited to model currents along a complex coastline, such as the fjord systems in Norway. Several earlier studies used FVCOM for aquaculture related challenges.^39−42^ In this study, FVCOM’s unstructured grid was used to refine the model resolution at an aquaculture site to resolve in detail the dispersion and dilution of deltamethrin post treatment. In the dispersion model, deltamethrin is treated as a passive tracer that is mixed into the water masses, and that does not affect the density of the water. In the Eulerian formulation used in FABM, the chemical is treated as concentrations in the model cells and is given in the model output directly. In the dispersion model, chemical degradation of deltamethrin is not considered since it is assumed to decrease the concentration at a much lower rate than the dilution in the water masses (e.g.,^43^ report a half-life in the water of 17.9 days). Similar dispersion modeling of deltamethrin after bath treatment in fish cages (using a Lagrangian particle tracking model) has previously been performed by Parsons et al.^9^ Concentrations, depth, and delousing parameters were set to be as realistic as possible. For example, seven out of 10 pens were deloused sequentially over 3 days, and the pens were set to be 10 m deep during delousing. More details about the dispersal modeling (assumptions, simulation details and domains, etc.) are found in the SI.

### 2.4 Statistical Analyses

Statistical analyses were performed using RStudio (version 0.99.491). The methods are described in the SI.

## 3 Results

### 3.1 Exposure of *P. borealis* to Short (1 h) Pulses of Deltamethrin

#### 3.1.1 Physical Conditions

The average seawater temperature was 6.4 °C (SD: 0.05). Average salinity was 34.3 ‰ (SD: 0.2), and the average oxygen saturation was 100% (SD: 0.5) throughout the experiment.

#### 3.1.2 Shrimp Behavior and Mortality

There was no significant difference in behavior or mortality between the control and the low and middle treatment doses (*p* < 0.005; Figure S5). However, in the experiment with highest concentration of deltamethrin (1/1000 of treatment dose; 2 ng L^–1^ deltamethrin), shrimp began to swim significantly more (10%, *p* = 0.01) immediately after the first pulse and then started to lay down. The shrimp laying down were immobilized and did not recover or react to stimuli and were therefore counted as dead. At the end of the three-pulse exposure (day 4), there was 80% higher mortality in this treatment compared to the control (*p* < 0.005). Due to animal welfare concerns, the highest treatment was ended after day 4. No mortality was observed during the 14 day recovery period in middle and low treatment doses or in the control.

#### 3.1.3 Sublethal Effects/Biomarker Analyses

**Figure 1:**
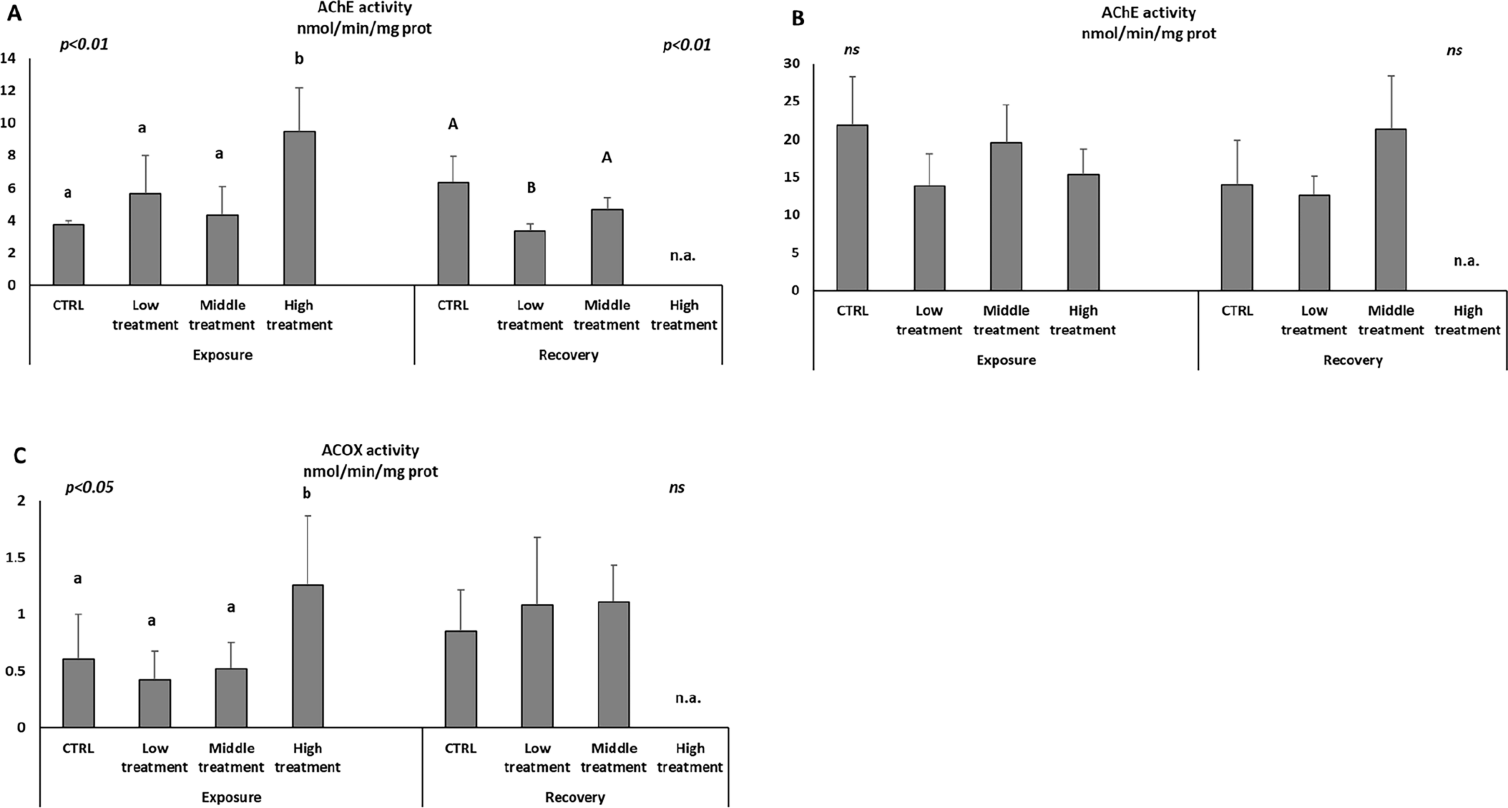
Activities of acetylcholinesterase (AChE) in gills (A) and muscles (B) and acyl CoA oxidase activity (ACOX) in digestive glands (C). Lowercase letters indicate significant differences between means of groups at the end of exposure time (end of the three-pulse exposure (day 4)); capital letters indicate significant differences between means of groups at the end of recovery time (14-day recovery period (day 18)). Data are given as mean values ± standard deviations, *n* = 15, ns = not significant variations, n.a. = not available.

There was a difference in AChE activity between gill and muscle tissues. The activity was significantly increased in gills of organisms exposed to the high deltamethrin treatment after the exposure period (day 4), while no significant effect was detected in the other treatments (Figure 1A). At the end of the recovery period a significant inhibition of AChE was observed in organisms exposed to low deltamethrin concentrations (Figure 1A). No clear trend was observed for AChE activity in the muscle samples, and the variability in results at each treatment and exposure period was high (Figure 1B).

A significant induction of the acyl CoA oxidase activity (ACOX) was observed only in shrimp exposed to high deltamethrin treatment after the exposure period (day 4). There were no significant variations observed for this enzyme at the end of the recovery period (Figure 1C).

The antioxidant response in shrimp was affected and oxidative damage induced. After the exposure period (day 4), deltamethrin caused a significant induction of TOSC toward peroxyl radicals (ROO^•^) in shrimp exposed to low and medium concentrations (Figure S6A) and a significant depletion of TOSC toward hydroxyl radicals (^•^OH) in those treated with the highest dose (Figure S6B). No variations in oxidative status were observed after the recovery period (Figure S6A,B). Malondialdehyde content did not exhibit any change in exposed or recovered shrimp under any experimental conditions (Figure S6C).

### 3.2 Field Sampling of Sediment and Water

#### 3.2.1 Calibration of the Passive Samplers

The polymer–water partition coefficient *K*_pw_ was determined in the laboratory for deltamethrin (Table S3; as well as for cypermethrin and diflu- and teflubenzuron (SI)). The differences in log *K*_pw_ estimated from the experiment with ultrapure water and from the cosolvent method were not significantly different (*p* < 0.005). For the cosolvent method (Figures S2 and S3), we used a simple linear regression between log *K*_pw_ values and the methanol content of the solution (on a mol/mol basis). Other models may be used for this, e.g., ref (34), but there is no strong basis for selection of one model over the other. The log *K*_pw_ values for deltamethrin were 5.86 and 5.45 for AlteSil and SSP silicone rubber, respectively. For cypermethrin, these were lower and 5.45 and 4.82, respectively. Values obtained through the cosolvent method were slightly higher than those with ultrapure water only. Log *K*_pw_ values for diflubenzuron were 2.00 (se = 0.20) and 2.32 (se = 0.21) for SSP and AlteSil silicone, respectively. For teflubenzuron, these were 2.95 (se = 0.19) and 3.33 (0.19), respectively.

#### 3.2.2 Field Concentrations of Deltamethrin in Water

Due to varying current conditions, the total exposure of PAS to deltamethrin may vary between a few hours to a theoretical maximum of time between beginning of delousing and time for collection of samplers (3.5 days). The length of PAS exposure to deltamethrin is an important factor during concentration calculations. The lowest concentration calculated in the present study was 11 ng L^–1^ (Table 1) at a 120 m distance from the nearest deloused pen. This is based on the assumption that deltamethrin was continuously present for 3.5 days after the beginning of the delousing. However, since currents vary and change directions, this is not a likely scenario. It is more likely that shorter exposures (hours) took place. This would lead to higher concentrations. Table 1 shows several scenarios since the exact time of exposure is not known. The analytical uncertainty is estimated at 50%.

**Table 1:** Calculated Concentrations (ng L^–1^) in Seawater at 3–5 m and 10–14 m Depth, Depending on Duration of the Exposure to Deltamethrin^a^

days of exposure	B–3–5 m	B–10–14 m	C–3–5 m	C–10–14 m	D–3–5 m	D–10–14 m	E–3–5 m	E–10–14 m
0.5	143	130	140	217	74	198	364	225
1	72	65	70	108	37	99	182	112
2	36	33	35	54	19	49	91	56
3.5	20	19	20	31	11	28	52	32
estimated distance (m) to deloused pen	45	45	105	105	120	120	15	15

a Sampler A (inside deloused pen) was lost in the field and therefore not included here.

#### 3.2.3 Field Concentrations of Deltamethrin in Sediment

At site 1, where 270 g of pure deltamethrin was used during delousing, concentrations in sediment ranged from 0.03 to 0.19 ng g^–1^ dw (Table S4). All samples at site 2 had concentrations < LOD, where 175 g of pure deltamethrin was used during the last delousing event (Table S4).

### 3.3 Model Results

**Figure 2:**
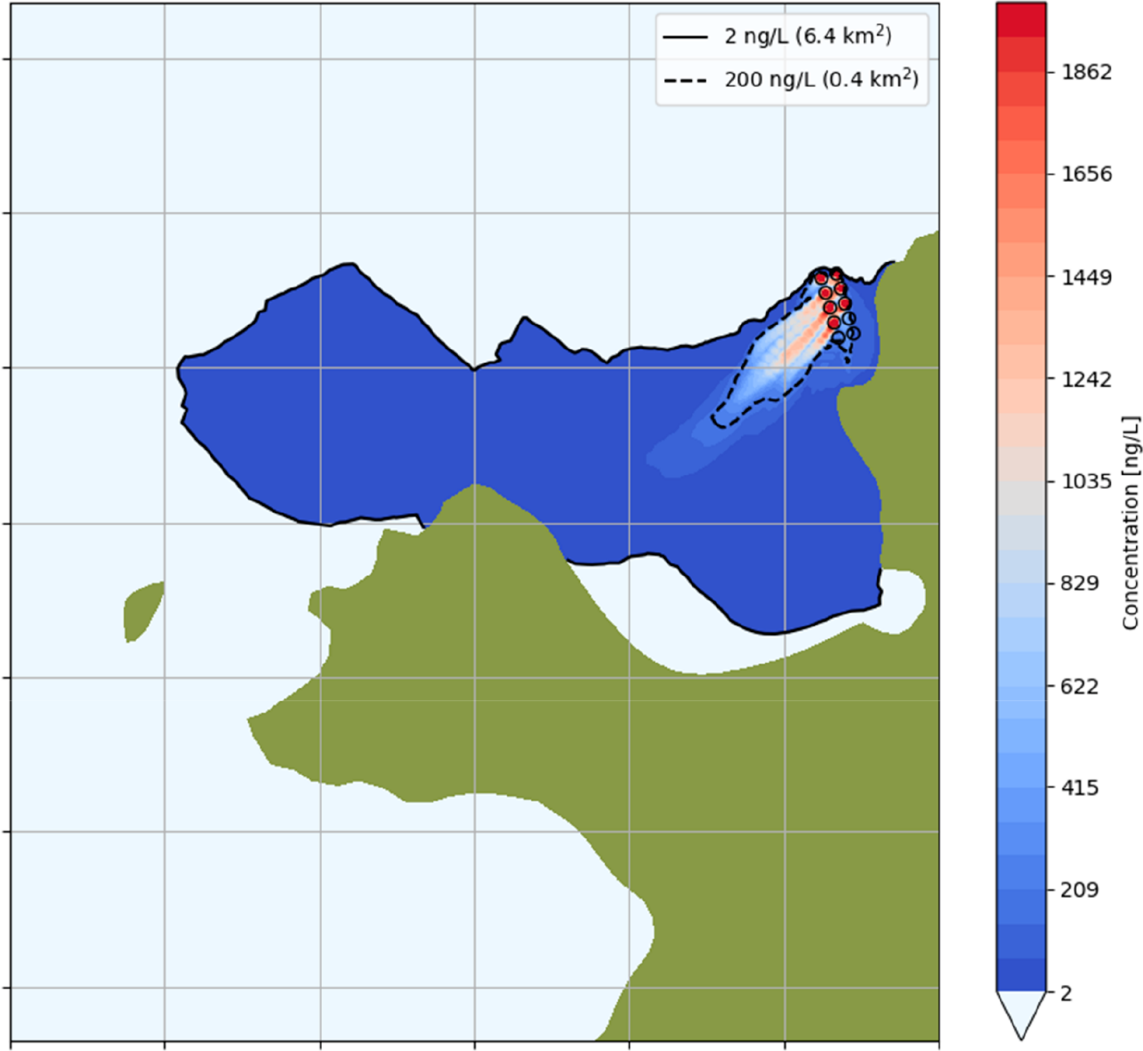
Model results showing the spreading of deltamethrin during the simulated delousing operation of seven cages. The colors indicate the maximum concentration within the water column during the entire simulation period (7 days). Contours of 2 ng L^–1^ (black solid line) and 200 ng L^–1^ (black dashed line) are plotted separately. Gridlines (gray) are spaced 1 km apart to indicate distance.

As the treatment water is released from each cage, the resulting plume will be transported by currents and diluted by mixing with the ambient water. Here, the plume is defined as the water with concentrations of deltamethrin above 2 ng L^–1^, since this was the lethal concentration to the shrimp derived in the laboratory experiments. The dilution is rapid, but since the lethal concentration for shrimp of 2 ng L^–1^ is only 0.1% of the treatment dose (2000 ng L^–1^), the plume can travel a significant distance away from the release site before being diluted to sublethal concentrations. The maximum concentration reached in the water column throughout the entire simulation is shown in Figure 2, indicating that lethal concentrations can spread approximately 4–5 km away from the release point, covering an area of up to 6.4 km^2^ (Figure 2). Figure S8 shows an accumulated exposure time of concentrations above 2 ng L^–1^ of the simulated delousing event, revealing that it is possible that lethal concentrations for shrimp can be present in the water column in some areas for approximately 35 h. By delousing each cage sequentially with 12-h intervals, there is no overlap of the plumes from the individual cages in these simulations. Examples of snapshots of maximum concentration in the water column 1 h after release from cages are shown in Figure S9.

## 4 Discussion

### 4.1 Acute Toxicity and Sublethal Toxicity

In this study, a repeated exposure of 1 h/day for three consecutive days to 1000 times diluted treatment concentration (i.e., 2 ng L^–1^) caused 80% lethality in shrimp. These results are comparable to those from a study^15^ where adult *P. borealis* showed up to 100% mortality after 2 h of exposure to 6 ng L^–1^ deltamethrin. Furthermore, Bamber et al.^18^ documented the high mortality of adult *P. borealis* exposed to a nominal concentration of 2 ng L^–1^ deltamethrin for 24 h. This exposure triggered an immediate increase in swimming, followed by reduced intensity, leaving all shrimp either moribund or dead. Bechmann et al.^16^ furthermore showed that shrimp larvae exposed for 2 h, 1–3 days post hatch were very sensitive to 2 ng L^–1^ deltamethrin, meaning that a 1000-fold dilution of the treatment dose of deltamethrin has a severe impact on shrimp.

High sensitivity to deltamethrin has also been documented for other crustaceans, such as European lobster *Homarus gammarus* (Linnaeus, 1758) and American lobster *Homarus americanus* (Edwards, 1837). The 1 h LC50 value for larvae is estimated to be 2.6 ng L^–1^ and 3.4 ng L^–1^, respectively, and for adults, 19 ng L^–1^.^9,26,43^ Other studies have reported varying sensitivity in crustaceans, from the very sensitive mysid shrimp (*Mysis sp*. (Latreille, 1802; 13.9 ng L^–1^)^26^ to less sensitive amphipod species with a 1 h LC50 of 187 ng L^–1^.^7,26^

The different studies performed during recent years clearly demonstrate that there are species-specific differences in sensitivity to deltamethrin among crustaceans. Bechmann et al.^16^ suggested that comparisons between studies may be partly hampered by differences in experimental designs (e.g., static vs flow through) or exposure vs recovery times in the lobster and shrimp experiments (1–2 h exposure 95 h to 14-day recovery) compared to chameleon and grass shrimp (1 or 24 h exposure and 24 h recovery). Delayed effects, including mortality, have been observed for both *P. borealis* exposed to the delousing agent Paramove (hydrogen peroxide) and oil,^44,45^ emphasizing the importance of testing organisms for several days postexposure to detect delayed effects. Furthermore, the difference in the toxicity may be due to testing the actual formula (Alpha Max) and not only the active ingredient deltamethrin as in some studies: additives in formulations are thought to influence the properties of the active ingredient (e.g., solubility, toxicity, fate, persistence).^46^ Other environmental factors may also influence the sensitivity of pharmaceuticals used for delousing and should be considered in the future. Bechmann et al.^47^ showed additive effects of diflubenzuron, an infeed delousing pharmaceutical, and ocean acidification/warming on the mortality of adult *P. borealis*.

Sublethal effects were observed in the current study in shrimp exposed to the high deltamethrin treatment. Few studies have investigated the effect of pyrethroids, e.g., deltamethrin, on AChE, and these generally indicate a decrease of this enzymatic activity in different fish and shrimp tissues after administration of sublethal concentrations.^48−50^ An increase in AChE activity has been reported for polychaetes (*Nereides diversicolor* (Müller, 1776)), mussels (*Mytilus galloprovincialis* (Lamarck 1819)), and amphipods (*Ampelisca brevicornis* (Costa, 1853)) exposed to different concentrations of tamoxifen (a pharmaceutical);^51^ furthermore, the biphasic responses of AChE in bivalves have been reported, with an induction at low concentrations and a decrease at a higher concentration of the tested contaminant.^52^ Pyrethroids can modulate AChE activity through the increased biosynthesis of the soluble isoform and the decrease of cholinesterasic membrane functionality. An increased neurosynaptic effect can represent a transitory mechanism to overcome an AChE inhibition, explaining upregulated AChE expression during oxidative stress and activation of kinase signaling cascade. However, the increase in AChE activity in the presence of reactive oxygen species (ROS) may cause a reduction of cholinergic neurotransmission efficiency and neurological dysfunctions since the essential acetylcholine is rapidly hydrolyzed in the synaptic cleft. In this sense, impairments in the shrimp’s normal behavior, increased swimming followed by laying down, and mortality observed in this study for the organism exposed to the high treatment may be linked to such a neurotoxic response. Bamber et al.^18^ also experienced increased activity first, followed by normal activity, before activity declined and shrimp were found to be either dead or moribund. The variable and more limited response of AChE in muscle tissues would reflect that the route of exposure of shrimp to deltamethrin is mostly via the gills.

A marked and significant induction of the ACOX in the high deltamethrin treatment indicates the responsiveness of peroxisomes to this agent. Several field and laboratory studies have shown that certain environmental contaminants, including pesticides, can induce peroxisomal proliferation in fish, mussels, and crustaceans.^53^ Peroxisomal proliferation is a cellular process, characterized by changes in peroxisome morphology and metabolism, with induction of enzymes involved in fatty acid oxidation, such as ACOX. During peroxisome proliferation, the induction of peroxisomal proteins is heterogeneous, with increased activity of enzymes involved in various aspects of lipid homeostasis. Pyrethroids possess agonistic activities toward human and/or mouse nuclear receptors PXR, CAR, and PPARa, supporting their mode of action as peroxisomal proliferators. Deltamethrin was able to induce PPARγ in murine NIH-3T3 and monkey COS-7 cells,^54,55^ but in contrast to other PPARγ activators, it does not induce the adipocytes differentiation, with a consequent decrease in cellular lipid content.^55^

Rates of ROS production can be increased by the presence of pesticides, a process often modulating the occurrence of cell damage.^56−58^ In our study, the slight induction of TOSC-ROO^•^ observed in shrimp exposed to low and middle treatments indicates a counteractive capacity of these organisms toward the deltamethrin-induced pro-oxidant challenge. On the contrary, the lower capability to neutralize OH after exposure to the high dose is predictive of enhanced oxidative toxicity. Even though a certain variation of malondialdehyde levels could have been expected, our results are similar to those of Dorts et al.,^59^ showing that deltamethrin acts in different ways in shrimp tissues, with a significant induction of lipid peroxidation in gills and not in digestive glands.

The sublethal effects investigated herein occurred at concentrations that were similar to lethal concentrations. Hence, lethality would have been a “good” alone measurement of deltamethrin effects on shrimp in the present study. However, other biomarkers not tested within the present study may give different results.

### 4.2 Deltamethrin Field Concentrations and Modeled Impact Zones

In this study, the field measurements of deltamethrin in water and sediment after delousing revealed detectable levels in both the surrounding water and the adjacent sediments. The relatively low concentrations of deltamethrin in sediment samples at site 1 (0.03 to 0.19 ng g^–1^ dw of deltamethrin) and the lack of detection at site 2 do not necessarily reflect the maximum levels occurring. Sediment samples are taken at a single point in time and space, and concentrations are often patchily distributed. The discrepancy between the two sites could be the result of differences in the amount of deltamethrin used, weather conditions, topography, depth, sediment composition, and currents. Both sites experienced high wind events between delousing and sampling, which might have increased the spreading and dilution. The average water temperatures were comparable at both sites (i.e., 5–6 °C in December and 3–4 °C in March^60^), but the differences in depth (20–50 m at site 2 and 50–100 m at site 1) might lead to a higher wind driven impact on currents and turbidity at site 2. Hence, less wind driven impact of site 1 might explain why deltamethrin was detected here although surface current is stronger (0–20 cms^–1^) compared to site 2 (0–10 cm s^–1^).^60^ On the other hand, a larger water depth should in theory lead to more dispersal during the sedimentation phase. Disturbance of the top sediment layer in the sediment grab due to “dilution” with deeper sediment (where deltamethrin is less likely to be absorbed) could also explain lower concentrations.

Strachan and Kennedy^46^ reported that the estimated half-life of deltamethrin was 17.9 days in water and 45.2 days in aerobic sediment, values similar to those reported by Meyer et al.^61^ and corresponding to 11.7–44.6 days in sediment for aerobic water–sediment systems. Considering the short half-life of deltamethrin, higher concentrations during the weeks before sampling cannot be excluded as samples were collected 5–6 weeks after the delousing. When the toxicity of deltamethrin was evaluated by SEPA for the Scottish market, the proposed predicted no effect concentration (PNEC) for organisms was 0.33 ng g^–1^ dw in sediments, and a study of sediment exposures of amphipods to deltamethrin over 10 days documented an LC50 of 16 ng g^–1^ dw.^62^ Our field data were 80–500 times lower than 10-day LC50 for amphipods, but only 2–11 times lower than the suggested PNEC decided by SEPA. However, due to few samples and the time between delousing and sampling, the relatively low measured concentrations may not be representative for maximum levels present at the sites.

Log *K*_pw_ values for the PAS AlteSil (5.86) and SSP (5.45) measured in this study are significantly higher than those reported earlier for deltamethrin (4.70 and 4.37, respectively).^63^ However, it is generally acknowledged that differences in log *K*_pw_ between different types of silicone rubber are possible. These log *K*_pw_ between 5 and 6 confirm that these compounds are generally amenable to passive sampling with silicone rubber. Water concentrations of deltamethrin were calculated based on these uptake experiments and estimated exposure times. Theoretically, the PAS could have been exposed to a plume for only a few hours, or for up to 3.5 days, or to several plumes from different cages that were treated subsequent to each other. The longer the time of exposure is set in calculations, the lower the water concentrations will be. That is, the lowest estimated water concentrations in the present study (11 ng L^–1^) would require 3.5 days of exposure, which is an unrealistic length of exposure. A water concentration estimation based on 12 h is probably closer to a realistic scenario and provides water concentrations between 74 ng L^–1^ of deltamethrin (120 m distance from the deloused pens) and 364 ng L^–1^ (15 m distance to deloused pens). An even shorter time of exposure for the PAS is probably an even more realistic scenario, which then suggests higher water concentrations (around 150–700 ng L^–1^, 520–1457 ng L^–1^, and 1625–4553 ng L^–1^ deltamethrin for 6, 3, and 1 h exposure of PAS, respectively). Short exposure times to high deltamethrin concentrations is supported by the dispersal modeling results (Figures 2 and S8). The model results indicate that high concentrations may affect the area close to the pens, but with rather short exposure times. For example, concentrations around 1000 ng L^–1^ occur up to about 500 m away from the pens (Figure 2). However, since the field measurements and model simulations used in this study are not from the same site, the comparison should be done with care. The model results strongly indicate that deltamethrin will be transported away from the pens as plumes of rather high concentrations, suggesting that the PAS deployed near the farm are likely to be exposed to high concentrations over short periods of time. For concentrations of 2 ng L^–1^ corresponding to a 1000-fold dilution, the areas of maximum exposure time may be located several kilometers away from the release site (Figure 2).

The benefit of PAS versus a “traditional water sample” is that the PAS will be exposed for deltamethrin if deployed downstream from the deloused pens. A “bucket water sample” needs to be timed with a plume and speed of currents to sample actual concentrations. The risk of missing the deltamethrin plume is much higher compared to PAS, and hence, “false negatives” might occur. The current study was a pilot and a first-time using PAS to measure deltamethrin after delousing, and it shows that a short exposure time to high concentrations is most likely close to the pens. Therefore, a next step sampling should be performed farther away from the pens, and the exposure time of the PAS should be shortened. Even though PAS is a good option, sampling at an increasing distance with a greater radius away from the pen may make it gradually more difficult to “detect” the actual plume without using multiple sampling points.

To our knowledge, there is only one study that measured field concentrations of deltamethrin in seawater after delousing. Concentrations of 1 ng L^–1^ deltamethrin were measured in water sampled 1000 m from a fish farm in Canada 48 h after bath treatment release, while 10 ng L^–1^ was measured approximately 120 m from the farm.^27^ Dye releases simultaneous with delousing have been used to estimate distribution and dilution. Ernst et al.^64^ showed that cypermethrin concentrations were closely correlated to the dye concentrations, and the dye traveled 900–3000 m from the release point. A similar study on deltamethrin stated that concentrations above LC50 (13.1 ng L^–1^) for amphipods^43^ would occur 120 m from the release point.^27^ The current study reflects the Canadian study that shows that toxic concentrations in field (>2 ng L^–1^) can be measured 120 m away from the release point. The dispersal modeling in this study further confirms this and shows lethal concentrations to shrimp (2 ng L^–1^) 4–5 km away from the release point and in an area of approximately 6–7 km^2^. The concentrations > 2 ng L^–1^ last 1–2 h in most places, and up to 35 h in other areas. A similar dispersal modeling of deltamethrin Parsons et al.^9^ demonstrated considerably larger affected areas compared to the current study for their farm sites, covering a mean area of 21.1–39.0 km^2^. Differences might be due to the amount of deltamethrin used and variations in meteorological and oceanographic conditions between farm sites. The impact on nontarget species will therefore depend on the specific geographical region and weather conditions occurring at the time of treatment. Sævik et al.^12^ for example showed that locations within fjords have slower dissolution rates and larger impact zones compared to exposed locations off the coast, especially during summer.

It is important to discuss current findings in relation to underlying assumptions and limitations of the models and impact area. The model may overestimate the dispersal of deltamethrin and therefore the extent of the potential impact zones. For example, the model assumes that deltamethrin remains in the water and does not adsorb to organic matter for 24 h post-release. On the other hand, underestimation may also occur because of the assumption on one isolated treatment per farm. But farms can conduct treatments on multiple pens per day. Furthermore, the delousing agents can often be used “off label” in concentrations higher than recommended dosages, as shown at site 2. The combination of higher concentrations and multiple treatments coupled with varying geographical conditions can generate higher concentrations and larger zones of potential impact than indicated above. Last, coherent delousing of all farms in an area to avoid early reinfection of sea lice is common and could affect multiple areas within a fjord or region at the same time.

Field measurements in the vicinity of the fish farms after delousing are in line with the results from the oceanographic modeling that high concentrations of deltamethrin can be found close to the pens after delousing. Both field measurements and dispersal modeling show harmful concentrations for shrimp present at sufficient times and in several square kilometers after a delousing event.

### 4.3 Possible Consequences in the Field

The present study shows that deltamethrin is highly toxic to the northern shrimp *P. borealis.* A 1000-fold dilution (2 ng L^–1^) of the active ingredient in a prescribed dose for delousing salmon (2000 ng L^–1^) in Norway causes high mortality in shrimp, and the few surviving organisms experience sublethal effects. This is in line with previous studies on shrimp and other nontarget species. These results, combined with measured and modeled dispersion of discharged deltamethrin, indicate that toxic concentrations could reach several kilometers away from a treated salmon farm and remain in the environment long enough to cause severe impacts on nontarget organisms. Many salmon farms in Norway are placed in the vicinity of shrimp fishing areas according to maps available (https://kart.fiskeridir.no/), and this study therefore indicates that there can be a risk for the important northern shrimp in fjords after delousing with deltamethrin. Furthermore, it confirms, together with other studies, that relatively large areas around aquaculture facilities can be exposed to lethal concentrations of deltamethrin following treatments, which are likely to have widespread adverse effects on sensitive nontarget crustacean species. The defined protection zone of no release of bath chemicals 500 m from known shrimp fields in Norway might therefore not be sufficient to protect shrimp. The size of the impacted areas may vary and will however depend on the specific geographical and weather conditions occurring at the time of treatment.

## Supporting Information Available

Additional information on methods and results (PDF)
