# Supplementary material for: Acute and Sublethal Effects of Deltamethrin Discharges from the Aquaculture Industry on Northern Shrimp (Pandalus borealis Krøyer, 1838): Dispersal Modeling and Field Investigations
Source: Environ Sci Technol. 2023 Feb 24;57(9):3602–11. doi: 10.1021/acs.est.2c07459 (PMC9996817; doi:10.1021/acs.est.2c07459)
Supplement: Supplementary file 1 — es2c07459_si_001.pdf [file es2c07459_si_001.pdf]

Supplementary material; Acute and sub-lethal effects of deltamethrin discharges from the aquaculture industry on northern shrimp (Pandalus borealis Krøyer, 1838); dispersal modeling and field investigations

Maj Arnberg[a], Gro Harlaug Refseth[a], Ian John Allan[b], Maura Benedetti[c], Francesco Regoli[c], Luca Tassara[d], Kjetil Sagerup[d], Magnus Drivdal[d], Ole Anders Nøst[a], Anita Evenset[d], Pernilla Carlsson[e*]

[a]Akvaplan-niva, Pirsenteret, havnegata 9, 7010 Trondheim, Norway

[b]Norwegian Institute for Water Research (NIVA), Gaustadalléen 21, 0349 Oslo, Norway

[c]Department of Life and Environmental Sciences, Polytechnic University of Marche, 60 131 Ancona, Italy.

[d]Akvaplan-niva, Fram Centre, Hjalmar Johansens gate 14, 9007 Tromsø, Norway

[e]Norwegian Institute for Water Research (NIVA), Fram Centre, Hjalmar Johansens gate 14, 9007 Tromsø, Norway
Corresponding author: *Pernilla Carlsson

Table of content

1. Material and methods, additional information

1.1 Additional information on the investigation of the effects in P. borealis of exposure to short (1 h) pulses of diluted deltamethrin.

1.1.1 Collection of shrimps

1.1.2 Exposure scenarios, experimental setup and stock preparations

1.1.3 Effect parameters analysis of sub-lethal effects

1.2 Field sampling of sediment and water

1.2.1. Evaluation and validation of passive sampling technique and partitioning coefficient experiments additional information

1.2.2 Deployment of passive samplers and surface sediment collection additional information.

1.3. Chemical analyses of sediment and water samples and calculations of dissolved concentrations of deltamethrin in water

1.3.1. Sample preparation, clean-up of sediment and water samples.

1.3.2. Quality control

1.3.3. Calculation of PAS deltamethrin concentration

1.4 Oceanographic modelling

1.5 Statistical analyses

2. Results, additional information

2.1.2 Shrimp behaviour and mortality

2.1.3 Sublethal effects/biomarker analyses

2.2.2 Water laboratory experiments

2.2.3. Sediment field concentrations

2.3 Model results

3. References

1. Materials and methods, additional information

1.1 Additional information on the investigation of the effects in *P. borealis* of exposure to short (1 h) pulses of diluted deltamethrin in the Alpha Max ® formulation.

1.1.1 Collection of shrimps

Northern shrimps were collected with shrimp pots in fjords in northern Norway in October 2019. Fjords with no aquaculture activities were chosen for sampling. Shrimps were stored in tanks with constant flow of fresh sea water while onboard the ship. Collected individuals were visually inspected and transported to Akvaplan-niva's marine research station at Kvaløya, Tromsø, Norway. Shrimps were kept in tanks (600 L) supplied with a continuous flow (500 L/h) of filtered (60µm filter) and UV-treated bottom seawater (63 m depth) from the fjord Sandøysundet, adjacent to the research facility. All tanks were covered with a lightproof cover during acclimation and rearing. Temperature and oxygen saturation were monitored daily resulting in temperatures ranging from 2.0 to 5.1 C, a salinity of 34‰ and an O_2-saturation >10 mg/L. Shrimp tanks were daily checked for dead individuals. The animals were acclimated for a minimum of 2 weeks before experimental start.

1.1.2 Exposure scenarios, experimental setup and stock preparations

Water quality parameters (oxygen saturation, temperature and salinity) were monitored in the beginning and end of exposure, and during the post-exposure period using a hand-held and portable Oxyguard®. As the salinity in the intake water from the fjord is stable at 34 ‰ throughout the year, salinity was not monitored. During the exposure pulse, the header solution was pumped through Teflon tubing into the seawater inlet to each exposure aquarium by a peristaltic pump with a multi channeling system (model 520, Watson and Marlow, Cornwall, UK).

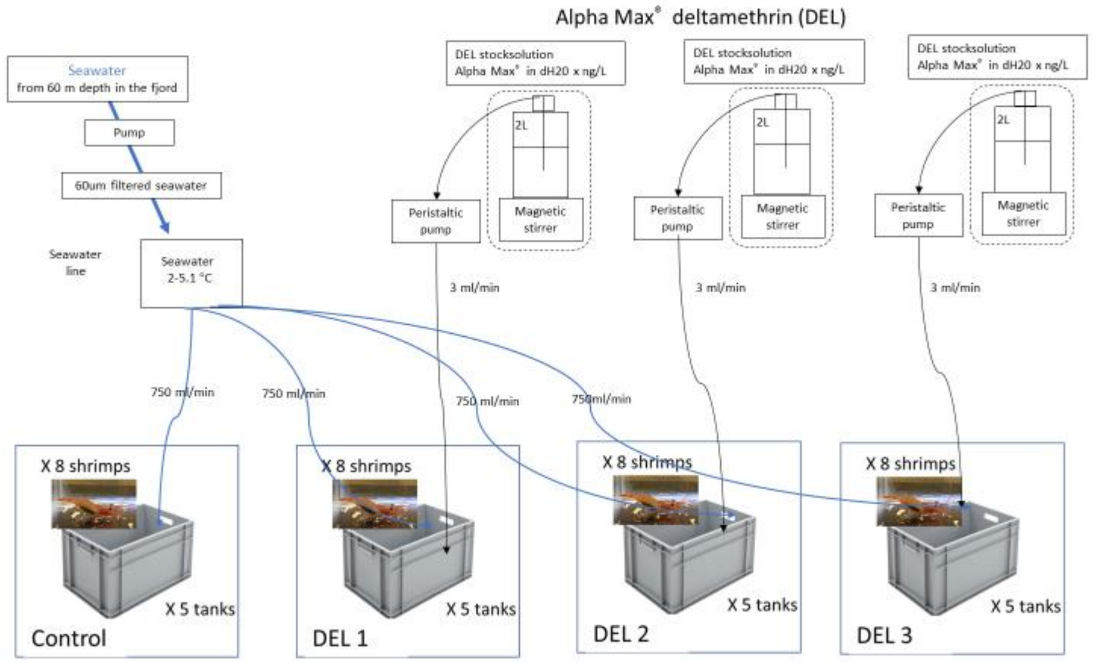

Figure S1. *Set-up of exposure system. Figure adopted to current conditions from the study from Renée Bechmann (NORCE).*

At the start of each exposure, the water flow was stopped, and 5 L water removed from each exposure tank, and replaced with 5L bath chemical in seawater at a concentration 9 times higher than the wanted

exposure concentration (i.e. diluted 9 times in the tank) to ensure the correct exposure concentration from the beginning. The experimental set-up is based on the system used by Frantzen et al. (2020). The water flow was then restarted, and, at the same time, two peristaltic multi-channel pumps were started, providing stock solution to each tank to ensure a constant concentration of the treatment chemicals throughout the exposure period with a flow of 3 mL/min. After 1 hour the peristaltic pumps were stopped, and the stock solution tubes removed from the tanks (i.e. start of recovery). Control tanks were subjected to the same procedure.

Three replicate tanks with 5 shrimp in each tank for each treatment were used, including control. All control tanks were handled the same way as the exposure tanks (i.e. pumped with clean water). Due to the relatively long exposure period, oxygen and temperature levels were also monitored in the middle of the experiment.

Stock solutions of deltamethrin were prepared in 2 L Schott bottles using the commercial formulations Alpha Max® and distilled water (Figure S1). The stock solutions were placed on magnetic stirrers. The stock solutions were used to achieve exposure concentration in the 2L headers, which contained 0.5ng/ml, 0.1ng/ml, 0.02 ng/mL of deltamethrin for the 1000 (high treatment), 5000 (middle treatment) 25000 (low treatment) dilutions respectively.

Table S1. *Description of the different treatments in the exposure experiment*

**Experiment, sub-lethal and lethal effects**				
**Name of treatment**	**Concentration DEL (ng/L)**	**Dilution of pharmaceutical recommended dose (2000 ng/L)**	**Pulses of DEL**	**Number of shrimp**
Control	0	-	Pulses of clean water	5 replicate tanks; 8 shrimp/tank
Low	0.0008	25 000	1h/day, 3 consecutive days	5 replicate tanks; 8 shrimp/tank
Middle	0.04	5 000	1h/day, 3 consecutive days	5 replicate tanks; 8 shrimp/tank
High	2	1 000	1h/day, 3 consecutive days	5 replicate tanks; 8 shrimp/tank

1.1.3 Effect parameters analysis of sub-lethal effects

All chemicals used for biochemical analyses were purchased from Sigma-Aldrich Co. (St. Louis, MO, USA).

Acetylcholinesterase activity (AChE)

Samples of gills and muscles were homogenized in 0.1 M trizma base, pH 7.2 containing sucrose 0.25 M and centrifuged at 10 000 g for 10 minutes. Obtained supernatants were spectrophotometrically assayed by the Ellman's reaction at 18 ± 1 °C, λ = 412 nm, ε = 13.6 mM cm^{-1} using acetylthiocholine and 5,5-dithiobis-2-nitrobenzoic acid (DTNB) as substrates. This colorimetric procedure is based on

the reaction of thiocholine (one of the products of enzymatic hydrolysis of acetylthiocholine) with 5,5-dithiobis-2-nitrobenzoic acid (DTNB, Ellman's reagent) forming a yellow product (5-mercapto-2-nitrobenzoic acid and its dissociated forms). The reaction medium was 0.1 M Trizma base (pH 7.4), DTNB 0.2 mM and acetylthiocholine 0.5 mM. After preincubation of DTNB and sample for 5 and 10 minutes for gills and muscles respectively, reactions were started by adding acetylthiocholine and readings were carried out for 1 minute at 412 nm (Ellman et al., 1961).

Activity of Acyl CoA oxidase (ACOX)

Samples of digestive gland were homogenized in 1 mM sodium bicarbonate buffer (pH 7.6) containing 1 mM EDTA, 0.1 % ethanol, 0.01 % Triton X-100 and centrifuged at 500 g for 15 min at 4 °C. The H_2O_2 production was measured in a coupled assay by following the oxidation of dichlorofluorescein-diacetate (DCF-DA) catalyzed by an exogenous horseradish peroxidase (HRP). The reaction medium was 0.5 M potassium phosphate buffer (pH 7.4), 2.2 mM DCF-DA, 40 μM sodium azide, 0.01 % Triton X-100, 1.2 U mL^{-1} HRP in a final volume of 1 mL. After a preincubation at 25 °C for 5 min in the dark with an appropriate volume of sample, reactions were started adding the substrates palmitoyl-CoA at final concentrations of 30 μM and 100 μM for Acyl-CoA oxidase (ACOX) and readings were carried out against a blank without the substrates at 502 nm (Small et al., 1985).

Antioxidant responses and oxidative damage

The antioxidant response and oxidative damage were analysed by measuring the total antioxidant capacity (TOSC assay towards peroxyl and hydroxyl radicals) and lipid peroxidation (malondialdehyde levels). For the total oxyradical scavenging capacity (TOSC) analysis, digestive glands were homogenized in 100 mM of potassium phosphate buffer (pH 7.5) containing NaCl (2.5 %), 0.1 mg mL^{-1} bacitracin and 0.008 TIU mL^{-1} aprotinin, 0.1 mg mL^{-1} leupeptin, 0.5 mg mL^{-1} pepstatin as protease inhibitors. After centrifuging at 100,000g for 70 min at 4°C, supernatants were collected and used for the analyses. The total oxyradical scavenging capacity (TOSC) assay measured the overall capability of cellular antioxidants to absorb different forms of artificially generated oxyradicals, thus inhibiting the oxidation of 0.2 mM α-keto-γ-methiolbutyric acid (KMBA) to ethylene gas (Regoli and Winston, 1999). Peroxyl radicals (ROO•) were generated by the thermal homolysis of 20 mM 2,2'-azo-bis-(2-methylpropionamidine)-dihydrochloride (ABAP) in 100 mM K-phosphate buffer, pH 7.4. Hydroxyl radicals (•OH) were produced by the Fenton reaction of iron-EDTA (1.8 mM Fe3+, 3.6 mM EDTA) and ascorbate (180 mM) in 100 mM potassium-phosphate buffer. Under these conditions, the different oxyradicals produced quantitatively similar yields of ethylene in control reactions, thus allowing the comparison of the relative efficiency of cellular antioxidants toward a quantitatively similar radical flux. Ethylene formation reactions were analysed at 12 min time intervals (total time: 96 min) by gas chromatographic separation and analyses by a detector (Agilent Technologies, Santa Clara, CA, USA) and the TOSC values were quantified by equation 1:

$$TOSC = 100 - (\int SA / \int CA \times 100), \qquad \text{(eq.1)}$$

where ∫SA and ∫CA are the integrated areas from the kinetic curves for samples (SA) and control (CA) reactions. For all samples, a specific TOSC (normalized to content of protein) was calculated by dividing the experimental TOSC values by the relative protein concentration contained in the assay.

Protein concentrations were measured according to the Lowry method, using bovine serum albumin (BSA) as standard (Lowry et al., 1951).

Malondialdehyde (MDA)

The shrimps' digestive glands were homogenized (1:3 w/v ratio) in 20 mM Tris–HCl (pH 7.4) and centrifuged at 3000 g for 20 min. A conjugation reaction was performed in 1 ml reaction mixture (45 °C, 40 min), containing 10.3 mM 1-metyl-2-phenylindole (dissolved in acetonitrile/methanol, 3:1), 32 % HCl, 100 µl water and 100 µl of sample or standard (standard range 0–6 µM 1,1,3,3-

tetramethoxypropane, in 20 mM Tris–HCl [pH 7.4]). Samples were finally cooled on ice, centrifuged at 15 000 g for 10 min and analyzed by spectrophotometer at 586 nm. MDA concentrations were determined as a function of the 1,1,3,3-tetramethoxypropane standard curve and expressed as nmol/g tissue (Benedetti et al., 2014).

1.2 Field sampling of sediment and water

1.2.1. Evaluation and validation of passive sampling technique and partitioning coefficient experiments

K_{pw} was measured for deltamethrin and cypermethrin using the co-solvent method (Booij et al., 2017; Pintado-Herrera et al., 2016; Smedes et al., 2009). As additional experiments, partitioning coefficients were also established for cypermethrin and the in-feed pharmaceuticals diflubenzuron and teflubenzuron. These measurements were undertaken for two types of passive samplers (SSP and Altesil™) and 8 water samples (n=16) simultaneously by equilibrating water/methanol solutions with deltamethrin and cypermethrin-spiked silicone rubber in laboratory-based batch experiments. The co-solvent methodology was first used to load the chemicals into the two types of silicone rubber (Booij et al., 2002) in a similar way to the spiking with PRCs (performance reference compounds). The compounds were added to methanol in a small vial together with the silicone rubber sheets cut to an appropriate size. The vial was shaken at 150 rpm on an orbital shaker and ultrapure water was added gradually over time to reach a 50:50 proportion of water and methanol over a one-week period. Silicone rubber sheets were then collected, dried with a lint-free tissue and place in a clean jar at -20 °C until use. All glassware was cleaned by heating in a furnace at 550 °C. Replicate assays with no methanol were conducted in 2 L-glass jars with just under 2 L of ultrapure water in contact with 50 mg of each of the silicone rubber. Experiments with a proportion of methanol (mol/mol) of 4 % (1 L solution and 100 mg silicone rubber), 9 % (1 L solution and 100 mg silicone rubber), 19 % (250 mL solution and 100 mg silicone rubber) and 45 % (250 mL solution and 2.2 g silicone rubber) were prepared. For the experiments with 0 and 4 % methanol, the glass wall of the jar was spiked with the two chemicals in order to shorten the time to equilibrium of these two assays. In short, the compounds were spiked in a small amount of methanol:water (20:80) that was subsequently added to the glass jars, the solution was rolled in the glass jar for some minutes. The solution was removed, and the bottle was rinsed twice with small amounts of ultrapure water to ensure no methanol was left in the 0 % methanol assays. The glass jars were then wrapped in foil and placed on an orbital shaker for 3 months at 150 rpm (slightly longer than planned due to covid-19 lock-down). Upon termination of the experiments, the solution (different volumes for different experiments) was placed in a decanter. In cases where the solutions contained significant amounts of methanol, water was added to dilute the methanol and ensure high recovery for the extraction of the chemicals. These samplers followed the same analytical protocol as the samplers exposed in field. Analyses and quantification were done as described in section 2.2.3.

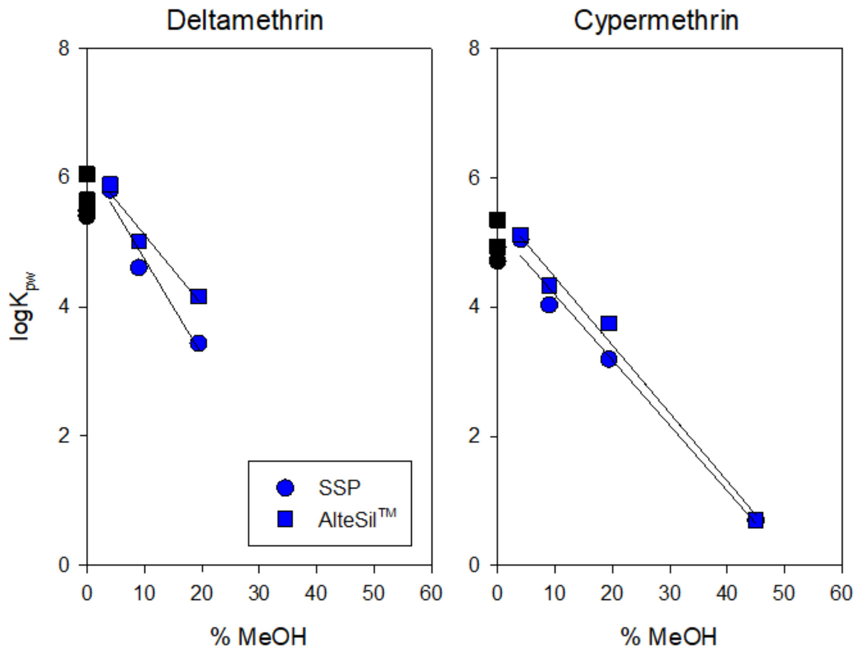

Figure S2. *Polymer-water partition coefficient (logK$_{pw}$) estimation for cypermethrin and deltamethrin obtained with the co-solvent method.*

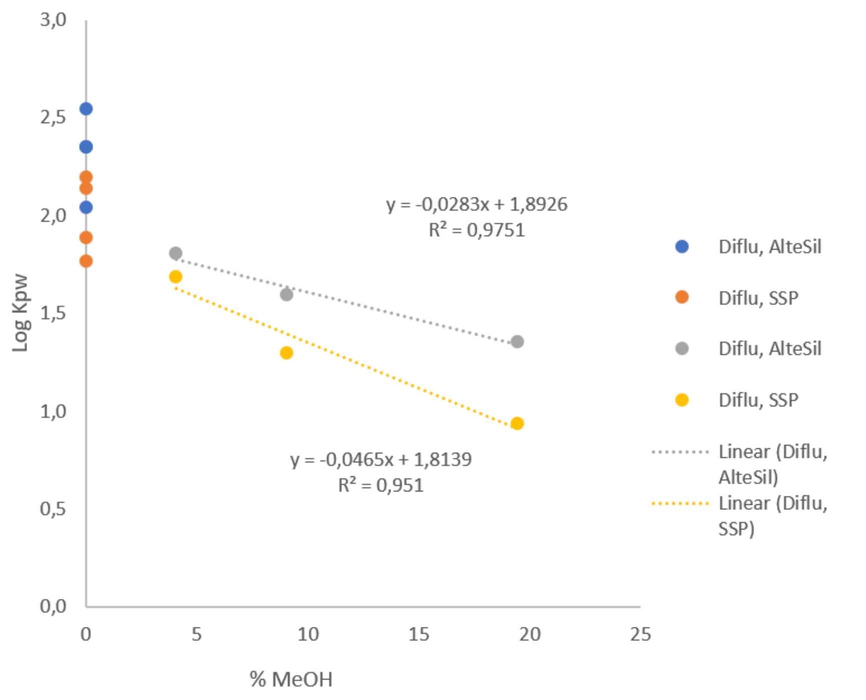

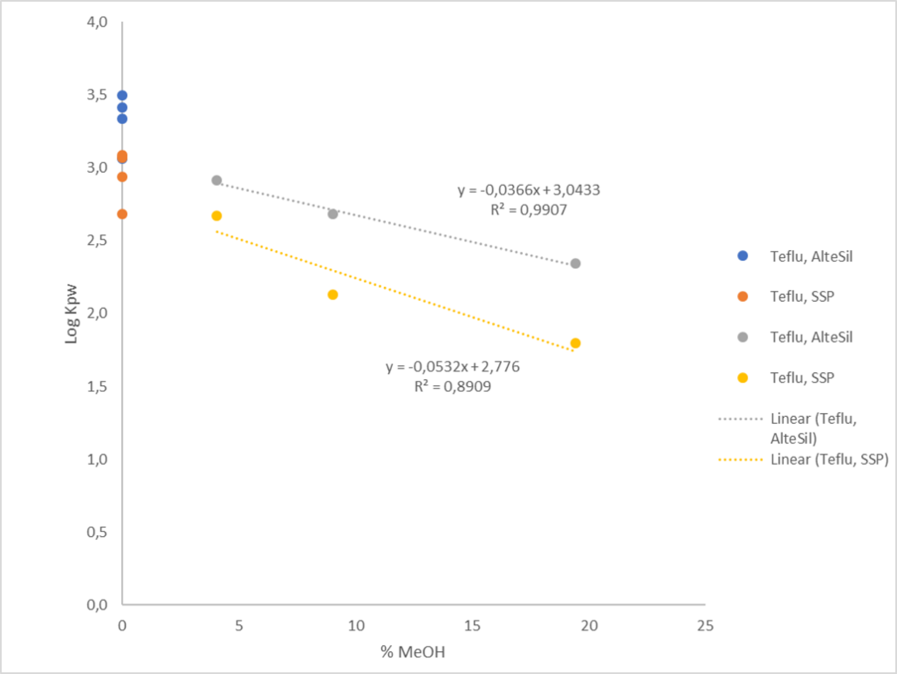

Figure S3. *Polymer-water partition coefficient (logK_{pw}) estimation for diflubenzuron (upper) and teflubenzuron (lower) obtained with the co-solvent method.*

1.2.2 Deployment of passive samplers and surface sediment collection

Site 1 is situated close to land and the soft bottom below deepens toward the fjord. The depth in the aquaculture area varies between 70-130 m. The fjord is 170 m deep. The dominating current at mid-depths is NW (315-345°) and the average speed is 3.0 cm/s (pers. communication Bjørn-Erik Bye, Akvaplan-niva 2020 and Havstraum (2022). Site 2 is also situated close to land, with soft bottom at

30-100 m depth in the aquaculture area. The fjord is 130 m deep and the dominating current at mid-depths is W-SW (240-270°) and the average speed is 3.0 cm/s (Havstraum 2022).

Before deployment, the passive samplers were spiked with the following performance reference compounds (PRCs); acenaphtene-d10, fluorene-d10, fenanthrene-d10, fluoranthene-d10, chrysene-d12, benzo(a)pyrene-d12).

Table S2. *Overview of collected water and sediment samples.*

**Site 1**				**Site 2**	
**Water-sample ID**	**Distance (m) to closest deloused pen**	**Sediment-ID**	**Distance (m) to closest deloused pen**	**Sediment-ID**	**Distance (m) to closest deloused pen**
A	Deployed in deloused pen but unfortunately lost	Sed-1		Sed-11	1
B	45	Sed-2	Inside deloused area	Sed-12	1
C	105	Sed-3	Inside deloused area	Sed-13	1
D	120	Sed-4	Inside deloused area	Sed-14	1
E	15	Sed-5	50-100*	Sed-15	100
		Sed-6	400	Sed-16	50-100*
		Sed-7	200	Sed-17	50-100*
		Sed-8	5-100*	Sed-18	50
		Sed-9	100	Sed-19	100-150*
		Sed-10	400	Sed-20	50-100*
				Sed-21	100
				Sed-22	100

*Some distances are given as range due to drift during sampling caused by high winds.

1.3. Chemical analyses of sediment and water samples and calculations of dissolved concentrations of deltamethrin in water

1.3.1. Sample preparation, clean-up of sediment and water samples.

All chemicals used for clean-up were of analytical grade and purchased from Sigma-Aldrich. Solvents were purchased from the following producers: dichloromethane, n-hexane and pentane from Rathburn (HPLC-grade), cyclo-hexane from Baker (Ultra Rezi-analyzed), ethyl acetate from Riedel-de Haen (Chromasolv LC-MS) and diethylether from Sigma-Aldrich (99,7 %).

Sediment samples: The sediment samples were clean-up and analyzed based on the method in Tucca et al (2017). Briefly, the samples were freeze dried and a subsample of 2-3 g was collected. D6-cyfluthrin was added as internal standard and the samples were extracted twice (ultrasonic bath) with 20 mL of dichloromethane and evaporated to 0.5 ml. Further clean-up was conducted with SPE-Florsil eluted with 20 % diethyl ether in iso-hexane followed by PSA and filtration through Costar spinex 0.2µm nylon filter.

Water samples: Internal standards were added to the passive samplers (d6-cyfluthrine and a mix of deuterated PAHs (d8-naphtalene, d10-biphenyl, d8-acenaphtylene, dibenzothiophene, d10-pyrene, d12-benz(a)anthracene, d12-perylene)) before they were extracted twice with pentane. The extracts were transferred to 1 ml ethylacetate/cyclohexane (80:20) and filtered through Spinex 0.2µm nylon. GPC was used to remove interferences. GPC-conditions: HPLC Agilent1260 Infinity equipped with

300 mm x 0.75 mm PL-gel column, 10 µm particle size eluted with a mix of ethyl acetate/cyclohexane (80:20).

All field samples were separated by an Agilent 7890B gas chromatograph (GC). This GC was equipped with two 15 m x 0.25 mm HP-5MS-UI columns with 0.25 µm film thickness and connected to a 7010B Triple Quadrupole mass spectrometer ((MS/MS) for detection and quantification of deltamethrin. One µl of sample extract was injected in splitless mode (injection temperature: 280°C). The oven temperature was 60°C and held for 1-minute, increased 40°C/min to 170°C followed by an increase of 10°C/min to 310°C and held for 4 min.
PAS were also analyzed for the reference PRCs by using GC-MS (Agilent Gas Chromatograph 7890A equipped with DB-5MS column, 30 m x 0.25 mm, 0,25 µm film thickness connected to Agilent 5975C inert XL Triple Axis detector run in SIM mode. The m/z transitions were: 250.7>172.0, 252.9>93.0 and 252.9>174 for deltamethrin and 198.9>170.1 and 226.9>76.9 for D6-cyflutrin. Further details can be found in Allan et al. (2013)

The clean-up procedure for diflu- and teflubenzuron for K_{pw} experiments followed the same procedure. More information about the analytical procedure can be found in Langford et al. (2014).

1.3.2. Quality control

One PAS field blank was exposed to air during the deployment. Laboratory blanks were analysed together with both water and sediment samples and all blanks were <LOD.
Two pre-spiked PAS (200 ng, i.e., in line with amount deltamethrin measured in the samples) were analysed parallel with the sediment samples from site 1 (133 % and 137 % recovery, respectively). The general, additive analytical uncertainty in the PAS analyses were estimated to be 40-60 %, considering analyses of both PRCs and deltamethrin. Three subsamples of the sediment samples, from site 2 were additionally spiked with 0.5, 2.5 and 50 ng/g, respectively and analyzed in parallel to make sure the analytical methods were working also for the quantification of sediment.

1.3.3. Calculation of PAS deltamethrin concentration

The uptake of deltamethrin in PAS are based on Rusina et al. (2010). In equation 1, where R_S is the in-situ sampling rate (L/d) and is estimated based on dissipation of spiked PRCs by the model from Rusina et al. (2010). β is a parameter that depends on e.g. temperature and flow-rate and was estimated based on R_S calculated from dissipation of the PRCs by plotting the retained PRC-fraction as a function of K_{sw} using non-linear least squares estimation (Booij and Smedes, 2010).

$$R_S=\beta K_{sw}^{-0,08} \qquad (1)$$

$$C_w = C_{PAS}/(R_s * t) \qquad (2)$$

The sampler water partition coefficient (K_{sw}) used in this study (6.2) to calculate C_W were obtained from experiments as described further up. K_{sw} is in general similar to K_{ow}, which is 5.9 for deltamethrin. Water concentrations are calculated according to equation 2. t is exposure time (d). The sampling rate estimated from the suite of PRCs (acenaphthene-d_{10}, fluorene-d_{10}, Phenanthrene-d_{10}, fluoranthene-d_{10}, chrysene-d_{12}, and benzo(a)pyrene-d_{12}) ranged between 1.8-5.9 L/d (log□ = 0.93 $L^{1.08}$ $kg^{0.08}$ d^{-1}; log□ se = 0.05) for an AlteSil silicone rubber with a mass of 16.5 g and surface area of 500 cm^2 deployed for 6 days.

Deltamethrin was distributed into the water after the PAS were deployed. The release of PRCs therefore occurred during six days, while the uptake of deltamethrin only occurred after the salmon delousing treatment began. The delousing took place over a few days, and the total exposure (days) of

deltamethrin to the PAS may therefore vary between a few hours to a theoretical maximum of time between beginning of delousing and time for collection of samplers (3.5 days).

1.4 Oceanographic modelling

During bath treatment, the cages are normally raised to about 10 m depth and covered by a tarpaulin before the de-lousing agent is added. After treatment the tarpaulin is removed, and the treatment water is released into the ambient water. To simulate this release in the model all the cells within a circle of the same horizontal size as the cage, and down to about 10 m, are initialized with concentrations equal to the treatment dose. The resulting plume of deltamethrin will then be transported and diluted in the model.
To model the dispersion at resolution down to about 15 m horizontally, two nested model domains are used. The outer model domain (Supplementary Figure S2A) covers a large part of northern Norway, while the inner model domain (Supplementary Figure S2B) covers parts of a fjord in northern Norway (Nordland), where the horizontal resolution around the fish farm is increased to about 15 m. A detailed illustration of the inner model grid close to the fish farm is shown in Supplementary, Figure S2C. To simulate a realistic delousing operation, the treatment procedure performed at the farm is followed as closely as possible. Hence, in the simulations, we have picked 7 of the 10 cages at the model site and released deltamethrin sequentially from each of these 7 cages at 12-hour intervals. The entire simulated operation then spans 3 days, and the simulation is run for 4 more days to ensure that concentrations of deltamethrin are negligible. To study the development of the discharge in time, the model output is saved at 10-minute intervals.

The model input builds mainly on oceanographical and hydrological data in the relevant area, and the input regarding deltamethrin is the amount used for the delousing at the site and timing for release and treatment of each cage. The hydrodynamic model FVCOM (Chen et al, 2003) has the possibility to adapt the grid size locally, which makes it ideal for modelling the currents along a complex coastline such as in Norway (ex. In Børve et al., 2021 and Nøst and Børve, 2021). Additionally, in this study we have exploited this flexibility of the model grid to increase resolution around the fish farm in order to study the detailed spreading pattern and dilution of the delousing chemical. The hydrodynamical model is coupled to a tracer module in FABM (Bruggemann and Bolding 2014), and the coupled FVCOM-FABM system used in this study is a state-of-the-art modelling tool that has been specifically developed and applied in several studies concerning the spreading of delousing chemicals (e,g. Refseth et al., 2016, 2019, Refseth and Nøst 2018).

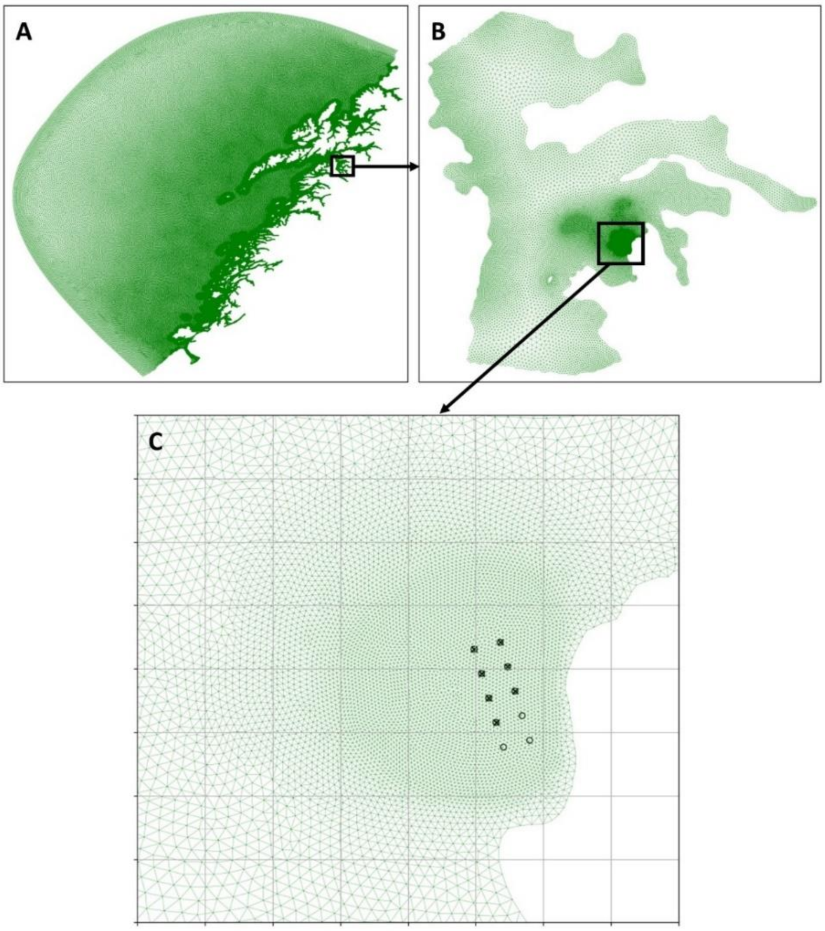

Figure S4: Grid of the Nordland Model (A), grid of the smaller model of the model fjord nested into the Nordland model (B) and a zoomed in picture of the grid close to the aquaculture farm (C). The fish cages are indicated by black circles, with crosses showing the cages used for the simulated delousing operation.

1.5 Statistical analyses

Levels of effect on shrimp observed after exposure of deltamethrin were compared statistically to levels of effect observed to untreated control handled in the same manner. Level of significance was set at $p < 0.05$. Mortality and behavior data analyses were performed in PAST v 17 (Hammer, 2001), using the Wilcoxon Rank sum test. Analysis of variance (ANOVA) was applied for all the other investigated shrimp parameters to test differences among experimental concentrations after experimental and recovery periods. Homogeneity of variance was checked by Cochram C and post-hoc comparison (Newman Keuls) was used to discriminate between means of values. Analysis of variance (2-way ANOVA) was applied to test differences among experimental conditions, experimental periods and interactions "exposure concentrations × periods" (level of significance at $p < 0.05$). All statistical analyses were performed using Rstudio (version 0.99.491).

2. Results

2.1.2 Shrimp behaviour and mortality

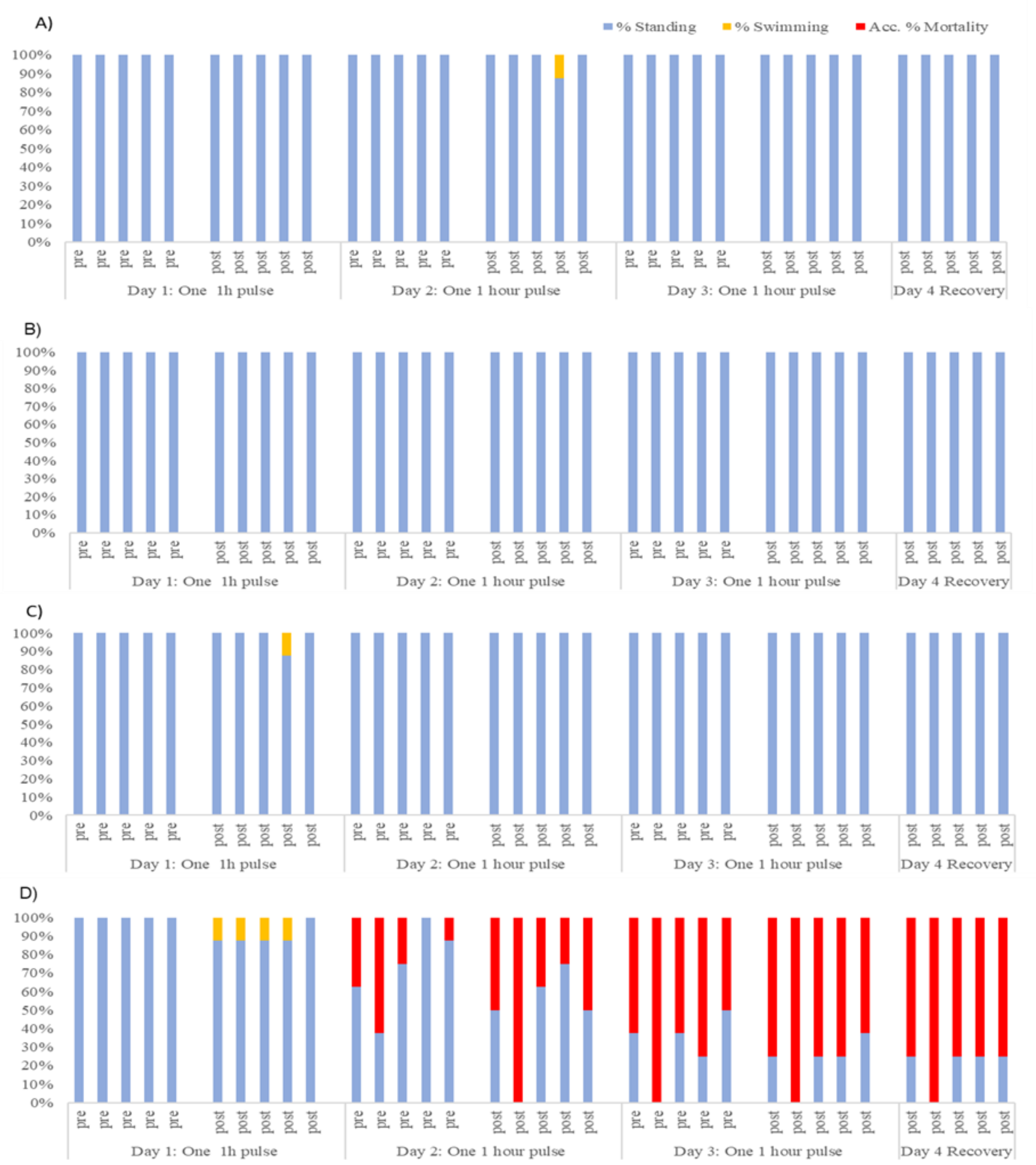

Figure S5. *Behaviour and mortality after 1h pulse/day over three days with deltamethrin including one day recovery. Behaviour and mortality were visually evaluated pre- and post-exposure during the exposure. Treatments: A) Control, B) Low treatment (0.0008 ng/L deltamethrin), C) Middle treatment (0.04 ng/L deltamethrin), D) High Treatment (2 ng/L deltamethrin). Number of shrimps per treatment were (n=8x3) at the start of the experiment. Light blue colour represents standing shrimp, orange swimming shrimp and red colour accumulated mortality.*

2.1.3 Sublethal effects/biomarker analyses

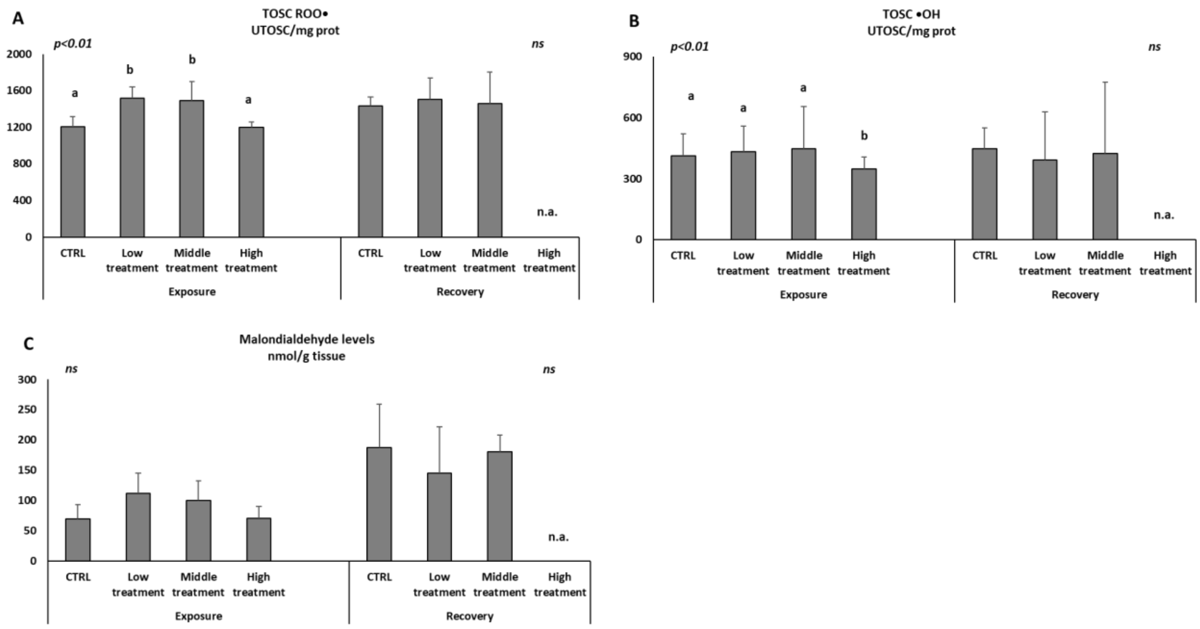

Figure S6. *Total Oxyradical Scavenging Capacity (TOSC) toward peroxyl (ROO•) (A) and hydroxyl (•OH) radicals (B) and malondialdehyde levels in digestive gland (C). Lowercase letters indicate significant differences between groups of means at the end of exposure time; capital letters indicate significant differences between groups. Data are given as mean values ± standard deviations,* n *=15. Ns= not significant variations; n.a.= not analysed.*

2.2.2 Water laboratory experiments

Table S3. *LogK_{pw} for deltamethrin in Altesil and SSP.*

	LogK_{pw} deltamethrin	
	0 % MeOH*	Co-solvent**
AlteSil™	5.86	6.20 (*0.29*)
SSP	5.45	6.23 (*0.40*)

*Relative percent difference of the replicate measurements of K_{pw} at 0 % methanol (MeOH) were 86 and 90 % for deltamethrin in AlteSil, and 20 and 49 % for SSP.
**standard error in brackets based on the linear regression of logK_{pw} with % methanol (mol/mol).

2.2.3 Sediment field concentrations

Table S4. *Sediment concentrations of deltamethrin, one month after the delousing event. Sed 1-10 are from site 1 (LOD varied between 0.02-0.1 ng/g dw) and sed-11-22 are from site 2 where no samples were >LOD (range 0.1-0.5 ng/g dw).*

**Sampling name**	**Site**	**Deltamethrin (ng/g dw)**	**Estimated distance to pen**
Sed-1	1	<LOD	0
Sed-2	1	0.15	0
Sed-3	1	<LOD	0
Sed-4	1	<LOD	0
Sed-5	1	0.10	50-100
Sed-6	1	0.03	400
Sed-7	1	0.19	200
Sed-8	1	0.13	50-100
Sed-9	1	0.03	100
Sed-10	1	<LOD	400
Sed-11-22	2	<LOD	0-300

2.3 Model results

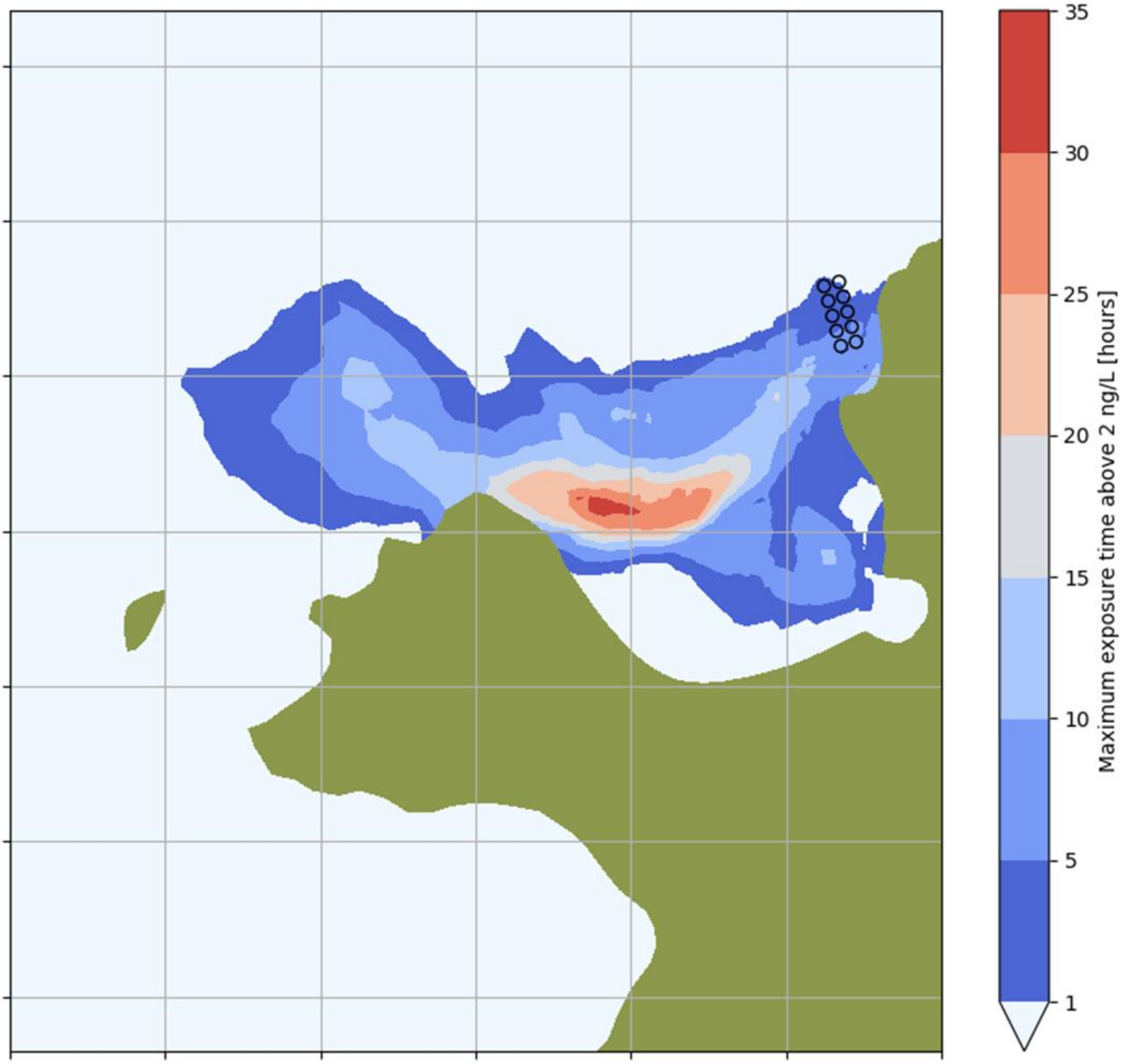

Figure S8. *Accumulated exposure time of concentrations above 2 ng/L during the entire simulation. Gridlines (grey) are spaced 1 km apart.*

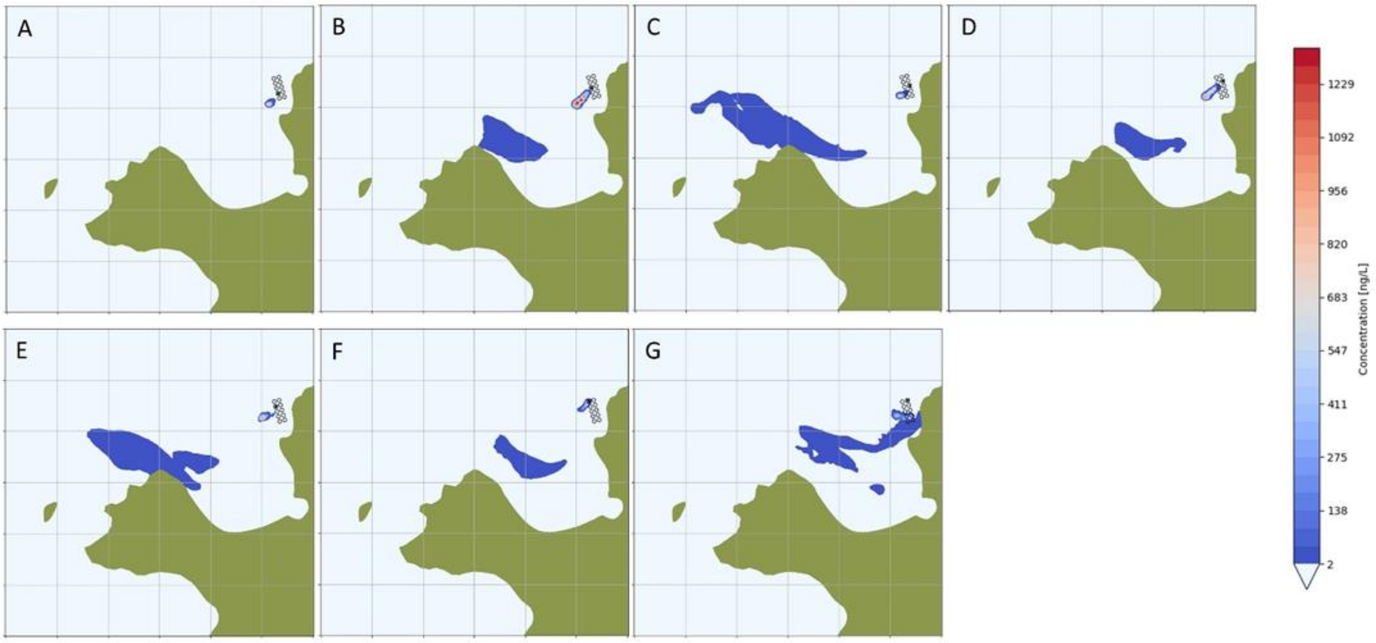

Figure S9: Snapshots of maximum concentration and spreading in the water column 1 hour after release from each cage; 1(A), 2(B), 3(C), 4(D), 5(E), 6(F) and 7 (G). Cages are shown to the upper right

3.References

Benedetti, M., Gorbi, S., Fattorini, D., D'Errico, G., Piva, F., Pacitti, D., Regoli, F. 2014. Environmental hazards from natural hydrocarbons seepage: Integrated classification of risk from sediment chemistry, bioavailability and biomarkers responses in sentinel species. Environmental Pollution. 185, 116-126.

Bruggeman, J. and K. Bolding, A general framework for aquatic biogeochemical models. Environmental Modelling & Software, 2014. 61: p. 249-265.

Børve E, Isachsen PE. Nøst OA (2021). Rectified tidal transport in Lofoten–Vesterålen, northern Norway", Ocean Sci., 17, 1753–1773, https://doi.org/10.5194/os-17-1753-2021

Chen, C., H. Liu, and R.C. Beardsley, An Unstructured Grid, Finite-Volume, Three-Dimensional, Primitive Equations Ocean Model: Application to Coastal Ocean and Estuaries. Journal of Atmospheric and Oceanic Technology, 2003. 20(1): p. 159-186.

Ellman G. L., Courtney K. D., Andres V. Jr., Featherstone R. M. 1961. A new and rapid colorimetric determination of acetylcholinesterase activity. Biochemical Pharmacology. 7, 88-95.

Frantzen, M., Bytingsvik, J., Tassara, L., Reinardy, H.C., Refseth, G.H., Watts, E.J., Evenset, A., 2020. Effects of the sea lice bath treatment pharmaceuticals hydrogen peroxide, azamethiphos and deltamethrin on egg-carrying shrimp (Pandalus borealis). Marine Environmental Research 159, 105007. https://doi.org/10.1016/j.marenvres.2020.105007

Havstraum (2022). www.havstraum.no. Current modeling, Akvaplan-niva.

Langford, K.H., Øxnevad, S., Schøyen, M., Thomas, K.V., 2014. Do Antiparasitic Medicines Used in Aquaculture Pose a Risk to the Norwegian Aquatic Environment? Environ. Sci. Technol. 48, 7774–7780. https://doi.org/10.1021/es5005329

Lowry O.H., Rosenbrough N.J., Farr A.L., Randall R.J. 1951. Protein measurement with the Folin phenol reagent. Journal of Biological Chemistry. 193, 266-275.

Nøst O.A. and Børve, E., 2021, " Flow separation, dipole formation, and water exchange through tidal straits", Ocean Sci., 17, 1403–1420, https://doi.org/10.5194/os-17-1403-2021

Refseth, H.G., Sæther, K., Drivdal, M., Nøst, O., Augustine, S., Camus. L., Tassara., Agnalt, A., Samuelsen, O.B., Miljørisiko ved bruk av hydrogenperoksid. Økotoksikologisk vurdering og grenseverdi for effekt. Akvaplan-niva rapport nr / report no 8200. 2016.

Refseth, G.H., Nøst, O.A., Miljørisikovurdering av Utslipp av Lusemidler Fra Lokalitet Bergkråa ‐ Hummer i Tysfjord Akvaplan-NIVA (2018), p. 33. 2018.

Refseth, G.H., Nøst, O.A., Evenset, A., Tassara, L., Espenes, H., Drivdal, M., Augustine, S., Samuelsen, O., Agnalt, A.-L., Risk assessment and risk reducing measures for discharges of hydrogen peroxide (H2O2). Ecotoxicological tests, modelling and SSD curve. Oceanographic modelling. (No. Akvaplan-niva report 8948-1). Akvaplan-niva. 2019.

Small G.M., Burdett K., Connock M.J. 1985. A sensitive spectrophotometric assay for peroxisome acyl-CoA oxidizing system in rat liver peroxisome. Biochemical Journal, 227, 205-210.

Tucca, F., Moya, H., Pozo, K., Borghini, F., Focardi, S., Barra, R., 2017. Occurrence of antiparasitic pesticides in sediments near salmon farms in the northern Chilean Patagonia. Mar. Pollut. Bull. 115, 465–468. https://doi.org/10.1016/j.marpolbul.2016.11.041
